# Nose and lungs: one way, one disease

**DOI:** 10.1186/1824-7288-38-60

**Published:** 2012-10-25

**Authors:** Davide Caimmi, Alessia Marseglia, Giovanni Pieri, Serena Benzo, Luca Bosa, Silvia Caimmi

**Affiliations:** 1 Department of Pediatrics of the University of Pavia, Fondazione IRCCS Policlinico San Matteo, Pavia, Italy

**Keywords:** Allergic rhinitis, Ashtma, Rhinosinusitis, United airways disease

## Abstract

It’s well established that asthma, allergic rhinitis and rhinosinusitis are three closely related disease. In pediatrics, these conditions represent a common issue in daily practice. The scientific community has recently started to simply evaluate them as different manifestations of a common pathogenic phenomenon. This consideration relates to important implications in the clinical management of these diseases, which may affect the daily activity of a pediatrician. The unity of the respiratory tract is confirmed both from a morphological and from a functional point of view. When treating rhinitis, it is often necessary to assess the presence of asthma. Patients with sinusitis should be evaluated for a possible concomitant asthma. Conversely, patients with asthma should always be evaluated for possible nasal disease, especially those suffering from difficult-to-treat asthma, in which an occult sinusitis may be detected. The medications that treat nasal diseases appear to be useful in improving asthma control and in reducing bronchial hyperresponsiveness. It seems therefore important to analyze the link between asthma and sinusitis, both in terms of clinical and pathogenic features, as well the therapeutic approach of those patients presenting with these diseases.

## Review

The link between asthma, allergic rhinitis and rhinosinusitis is well known and internationally accepted, while the precise concept of an united airways disease has been postulated only recently [1]. The atopic diseases of the nose and the lungs are, in fact, related to each other, and coexist in many patients, with a much higher frequency than expected, when simply considering the prevalence of each disease in the general population [2]. The immediate sign that the airways diseases coexist is given by the fact that upper respiratory tract infections are capable of exacerbating asthma, while rhinosinusitis has been identified as an independent risk factor for asthma development [3,4]. Even though this connection seems clear from a theoretical point of view, in daily practice, children presenting with nasal and respiratory symptoms are generally treated by two different specialists.

In Italy, the prevalence of current asthma, allergic rhinoconjunctivitis, and atopic eczema in 2006 was 7.9%, 6.5%, and 10.1% among children aged 6–7 and 8.4%, 15.5%, and 7.75% among children aged 13–14 years [5]. In children, atopic dermatitis represents the first step of the *atopic march*, followed by allergic rhinitis and asthma. We should start thinking now that the atopic march consists of a two-steps march, that is to say that eczema is the initial symptom of a change in the child’s immune system, followed by atopic respiratory symptoms, involving the nose, the nasal sinuses and the lungs [6]. The upper and lower respiratory tracts form a *continuum* and share many anatomical and histological properties and an important feature: the passage of air into and out of the lungs. Anatomically, the respiratory tree is classically distinguished into nose, mouth, pharynx, larynx, trachea and bronchi, which, in turn, are dicotomically divided until the respiratory bronchioles. These lasts, with their air cells, correspond to the respiratory portion reserved for pulmonary gas exchange. The upper respiratory tract begins with the nostrils and the mouth and ends with the larynx, while the lower tract goes from the larynx to the alveoli.

### Common triggers: not only allergy

The natural progression of respiratory allergy commonly starts from the upper respiratory tract and later spreads to the lower tract [7].

Several factors may trigger reflex airways reactions. Viruses and bacteria are responsible for infectious rhinitis, rhinosinusitis, bronchiolitis and pneumonia. In children, viruses are known to cause asthma exacerbation as well. Cigarette smoke and pollutants, atmospheric or professional, may damage the epithelium as well, and they may be responsible for a chronic inflammation. Tobacco smoke exposure in children is responsible of airways inflammation and infections, involving both the upper and the lower respiratory tract [8]. At last, even though asthma and allergic rhinitis are hypersensitivity reactions, they may also be triggered by drugs such as beta-blockers or aspirin, cold dry air exposure, and physical exercise [9].

It is clear, though, that allergens are potent stimuli of allergic rhinitis and asthma. Allergic patients suffer from a general inflammatory status interesting the whole respiratory tract and manifesting through different diseases, affecting the nose, the paranasal sinuses and the bronchi [10]. Nasal allergen exposure, in patients suffering from allergic rhinitis, may rapidly lead to a significant pulmonary allergic inflammation, even in subjects without a history of bronchial hyperreactivity [11]. Moreover, segmental bronchial challenge may induce nasal symptoms and inflammation in patients suffering from allergic rhinitis, as nasal challenge may induce a decline in respiratory functions [12,13]. The rates of indoor allergens such as dust mites or animal proteins are highly correlated with asthma, while the rates of outdoor allergens such as pollen are more often associated with rhinitis [14].

### A common pathogenesis

Several studies in recent years have tried to clarify what is beneath the link between inflammation of the upper and lower respiratory tract. Main theories include the nasal-bronchial reflex, the presence of inflammatory mediators draining from the upper to the lower respiratory tract, the role played by environmental factors, and the presence of a certain degree of hyperreactivity, interesting the epithelium of the entire respiratory tract [15].

In patients with asthma, the characteristics of the inflammation of the upper respiratory tract has common features with inflammation in the lung, such as the increased production of IgEs and the important role played by eosinophils, and cytokines (such as Interleukin-5) [16].

Various studies in recent years try to define and clarify the degree of inflammation in the lower respiratory tract in healthy patients, in patients with rhinitis, and in subjects with asthma and rhinosinusitis. The results of the various studies, however, have confirmed that the concentration of certain mediators (such as exhaled nitric oxide, or the amount of eosinophilic cationic protein, Inerleukin-5 and VEGF on sputum) show incremental values from healthy controls to patients with asthma and rhinitis and/or rhinosinusitis [17-21]. All these findings have been able to corroborate the theory of the “united airways disease”, considering therefore the upper and lower airways as a single anatomo-functional and pathogenic entity [15].

### The link between allergic rhinitis and asthma

Allergic rhinitis is the most common of all atopic diseases and it may develop at any age, even though in those patients who present during the first years of life signs of atopic dermatitis, it usually appears before the school age. Allergic rhinitis may be intermittent or persistent, depending on how long the symptoms last, and mild or moderate/severe, on the basis of the severity of the symptoms experienced by the patient [22]. Besides the classical symptoms of sneezing, rhinorrhea, and nasal obstruction, AR is characterized by impairment in the patients’ quality of life, and potentially by sleep disorders, emotional problems, impairment in activities, and social functioning [22,23].

Asthma is defined as a disease of reversible airway obstruction and is diagnosed by using measures of lung function and bronchial hyperreactivity. Main symptoms include dry cough, expiratory wheezing, chest tightness and dyspnoea, which are intermittently triggered by allergens, infections and airways irritants [24]. Small airway disease, defined as a reduction in forced expiratory flow (FEF) at 25–75% of the pulmonary volume and a normal spirometry, is suggested to be a marker of early allergic or inflammatory involvement of the small airways in subjects with allergic diseases and no asthma [25]. FEF25–75 seems to be significantly associated with bronchial hyperreactivity, and it has been proposed as an early marker of bronchial involvement in patients with allergic rhinitis who perceive nasal symptoms only [26,27]. In general, and this is especially true in children, there are even serum markers (such as Interleukin-23) able to highlight the severity of the respiratory disease, and the initial involvement of the small airways that characterizes the bronchial hyperresponsiveness [28,29].

Allergic rhinitis is considered as a risk factor for developing asthma, but it is possible that this term is not totally correct in the sense that this condition may represent an early stage of the united airways disease that may progress to asthma [25]. Approximately 19–38% of patients with allergic rhinitis have concomitant asthma and 30–80% of asthmatics have allergic rhinitis, although these data probably underestimate the phenomenon, as recent surveys found symptoms of rhinitis in 98.9% of allergic asthmatics and in 78.4% of non-allergic asthmatics [30]. Moreover, most of patients with allergic rhinitis show bronchial hyperreactivity, even though they do not present any clinical sign of lung function impairment nor of asthma and such a finding may represent a prognostic factor for further progression to asthma [31].

In those patients presenting with both allergic rhinitis and asthma at the same time, a significantly higher number of aeroallergen sensitizations may be detected than in those without asthma, and patients suffering from perennial rhinitis present an increased risk to develop asthma [32].

A small proportion of asthmatic patients present a severe form of asthma, which often requires a continuous and long-term treatment with oral corticosteroids to control symptoms [33]. Since difficult asthma is rare in childhood, when asthma is difficult to treat and poorly controlled, a special evaluation should include the review of diagnosis with accurate lung function and the evaluation of possible comorbidities.

### The link between rhinosinusitis and asthma

The term rhinosinusitis refers to the presence of inflammation within any of the four pairs of paranasal sinuses. Symptoms include nasal congestion, nasal discharge, nasal purulence, postnasal drip, facial pressure, hyposmia, cough, fever, halitosis, dental pain, ear fullness, and headache. The pathogenesis of this disease is poorly understood. The long-term consequences of chronic sinusitis may include lose of mucociliary clearance and of other physiologic mechanisms that normally maintain the relative sterility of the sinuses [34].

In children, paranasal sinuses disease is considered to an important risk factor for the development of lower respiratory tract diseases, and rhinosinusitis and asthma seem to be two different expressions of a common pathological process [33]. Recent progress in understanding the biology of airway disease has identified inflammation as the key to understand these diseases. Nevertheless, several other mechanisms that link the upper (nose, sinuses, larynx, pharynx, and trachea) and lower (bronchi and lungs) airway segments may be involved too [35].

Precipitants of asthma are generally also precipitants of sinusitis, and, therefore, the association of sinusitis with asthma exacerbations may be an epiphenomenon [34]. Rhinosinusitis coexists with asthma in 34–50% of patients [36]. Nevertheless, in patients presenting with asthma, the incidence of concomitant rhinosinusitis rise up to 84%, especially during asthma exacerbations [36]. The observation that asthma and rhinosinusitis coexist in patients at a higher frequency than would be expected from the prevalence of each in the general population provides a strong connection between the upper and lower airways [37]. Sinonasal disease in asthmatics appears to differ somewhat from that of the general population and the temporal sequence of disease and parallel inflammatory pathways involved suggest that they may be progressive manifestations of a common disease process [38]. It should also be pointed out that the characteristics of patients with chronic allergic rhinosinusitis differ from those of patients with non-allergic rhinosinusitis: in fact, the first group of patients are more frequently affected by asthma, and have a higher risk of having nasal polyposis, if compared to the second group [39].

### Therapeutic implications

There are several studies that have investigated whether improved control of sinusitis in a child with asthma also means improving asthma symptoms. Although further studies are certainly need to reach a definitive conclusion, different results seem to now provide encouraging data in this regard [40-42]. It was for example shown that patients with severe asthma are frequently bearers of the worst sinusal lesions, and that medical treatment of rhinosinusitis results in an improvement of clinical asthma and in a greater control of respiratory symptoms. Moreover, many papers have focused on the fact that an early allergen immunotherapy may not only reduce asthma symptoms but also improve the nasal disease as well [43,44].

The natural consequence of these considerations is that occult sinusitis may stand beneath many asthma exacerbations. Occult sinusitis is a clinically unrecognized sinusitis, which can be highlighted through imaging and is associated with a worse respiratory involvement. It is therefore important to exclude the presence of a sinusitis in patients with frequent exacerbations of asthma. Finally, it also seems reasonable to ask whether a prompt and early treatment of sinus infections in a patient with atopic eczema may affect the subsequent development of asthma: however, such a hypothesis, although some studies leave some hope, is not easy to demonstrate.

Another important issue that requires patients suffering from airways allergic disease to be properly treated, with a complete control of their respiratory symptoms, is the possibility of a surgical intervention. In fact, asthmatic patients or patients suffering from important nasal symptoms are often more at risk than the general population of developing complications during surgery, if their disease is not completely controlled. This implication seems even more important if we consider that surgical stress induces complex modifications in the hemodynamic, metabolic, neuro-hormonal and immune response of the individual, leading therefore to an increased risk not only of respiratory reactions, but also of infectious septic complications [45-47].

In conclusion, the therapeutic approach should focus not only on the control of the united airways disease to avoid respiratory complications, but also to provide a better quality of life in patients and to let them be comparable to the general population in everyday settings.

## Conclusions

Asthma, rhinitis, and sinusitis are three conditions frequently encountered during childhood, they have a common pathophysiological origin, and they may be associated to environmental stimuli and immunological predisposition [48]. Although further studies are certainly needed to clarify many aspects of this close relationship, pediatricians have to take into account, in approaching an asthmatic child, the possible nasal involvement, that we could call the “nasal asthma”. In clinical practice, therefore, a rigorous treatment of comorbid factors of ashtma, such as rhinosinusitis, could result in less asthma exacerbations, which will greatly improve the quality of life of difficult-to-control asthmatic patients. In conclusion, treating asthma means also treating the nose, while treating patients with nasal symptoms has to be associated to a proper lung function evaluation, since the nose and the lungs should always be considered as a unique entity.

## Competing interests

All Authors disclose any financial competing interest.

All Authors disclose any non-financial competing interest.

## Authors’ contributions

Each author (CD, MA, PG, BS, BL, CS) has contributed to writing the present papers and they all approved it in its final version.

## References

[B1] Passalacqua G Ciprandi G Canonica GW The nose–lung interaction: united airways disease Curr Opin Allergy Clin Immunol 2001 1 7 14 11964663 10.1097/01.all.0000010978.62527.4e

[B2] Marseglia GL Caimmi S Marseglia A Poddighe D Leone M Caimmi D Ciprandi G Castellazzi AM Rhinosinusitis and asthma Int J Immunopathol Pharmacol 2010 23 Suppl 1 29 31 20152076

[B3] Bousquet J van Cauwenberge P ARIA. Allergic Rhinitis and its Impact on Asthma. Position paper in cooperation with WHO J Allergy Clin Immunol 2001 108 S23 S35 10.1067/mai.2001.118891 11707753

[B4] The International Study of Asthma and Allergies in Childhood (ISAAC) Worldwide variation in prevalence of symptoms of asthma, allergic rhinoconjunctivitis and atopic eczema Lancet 1998 351 1225 1232 10.1016/S0140-6736(97)07302-9 9643741

[B5] Tozzi AE Armenio L Bernardini R Boner A Calvani M Cardinale F Cavagni G Dondi A Duse M Fiocchi A Marseglia GL Miraglia Del Giudice M Muraro A Pajno GB Paravati F Peroni D Tripodi S Ugazio AG Indinnimeo L Pediatric allergy and immunology in Italy Pediatr Allergy Immunol 2011 22 267 276 10.1111/j.1399-3038.2011.01157.x 21457333

[B6] Ciprandi G De Amici M Giunta V Marseglia A Marseglia G Serum Interleukin-9 Levels Are Associated With Clinical Severity in Children With Atopic Dermatitis Pediatr Dermatol 2012 in press 10.1111/j.1525-1470.2012.01766.x 22612419

[B7] Pedersen PA Weeke ER Asthma and allergic rhinitis in the same patients Allergy 1983 38 25 29 10.1111/j.1398-9995.1983.tb00852.x 6837893

[B8] Marseglia GL Avanzini MA Caimmi S Caimmi D Marseglia A Valsecchi C Poddighe D Ciprandi G Pagella F Klersy C Castellazzi AM Passive exposure to smoke results in defective interferon-gamma production by adenoids in children with recurrent respiratory infections J Interferon Cytokine Res 2009 29 427 432 10.1089/jir.2008.0108 19514840

[B9] Fontanari P Zattara-Hartman MC Burneet H Jammes Y Nasal eupnoeic inhalation of cold, dry air increases air way resistance in asthmatic patients Eur Respir J 1997 10 2250 2254 10.1183/09031936.97.10102250 9387948

[B10] Crimi E Milanese M Oddera S Mereu C Rossi GA Riccio A Canonica GW Brusasco V Inflammatory and mechanical factors of allergen-induced bronchoconstriction in mild asthma and rhinitis J Appl Physiol 2001 91 1029 1034 11509494 10.1152/jappl.2001.91.3.1029

[B11] Blaiss MS Rhinitis–asthma connection: epidemiologic and pathophysiologic basis Allergy Asthma Proc 2005 26 35 40 15813286

[B12] Braunstahl G Kleinjan A Overbeek SE Prins JB Hoogsteden HC Fokkens WJ Segmental bronchial provocation induces nasal inflammation in allergic rhinitis patients Am J Respir Crit Care Med 2000 161 2051 2057 10852787 10.1164/ajrccm.161.6.9906121

[B13] Braunstahl G Overbeek SE Kleinjan A Prins JB Hoogsteden HC Fokkens WJ Nasal provocation induces adhesion molecule expression and tissue eosinophilia in upper and lower airways J Allergy Clin Immunol 2001 107 469 476 10.1067/mai.2001.113046 11240947

[B14] Platts-Mills TAE The role of allergens in allergic air way disease J Allergy Clin Immunol 1998 101 S364 S366 10.1016/S0091-6749(98)70221-0 9500729

[B15] Ciprandi G Caimmi D Miraglia Del Giudice M La Rosa M Salpietro C Marseglia GL Recent developments in United airways disease Allergy Asthma Immunol Res 2012 4 171 177 10.4168/aair.2012.4.4.171 22754709 PMC3378922

[B16] Dorman SC Sehmi R Gauvreau GM Watson RM Foley R Jones GL Denburg JA Inman MD O'Byrne PM Kinetics of bone marrow eosinophilopoiesis and associated cytokines after allergen inhalation Am J Respir Crit Care Med 2004 169 565 572 10.1164/rccm.200307-1024OC 14656753

[B17] Miraglia Del Giudice M Marseglia GL Leonardi S Tosca MA Marseglia A Perrone L Ciprandi G Fractional exhaled nitric oxide measurements in rhinitis and asthma in children Int J Immunopathol Pharmacol 2011 24 Suppl 4 29 32 22032784 10.1177/03946320110240s407

[B18] Del Giudice MM Pedullà M Brunese FP Capristo AF Capristo C Tosca MA Leonardi S Ciprandi G Neutrophilic cells in sputum of allergic asthmatic children Eur J Inflamm 2010 8 151 156

[B19] Caffarelli C Calcinai E Rinaldi L Povesi Dascola C Terracciano L Corradi M Hydrogen peroxide in exhaled breath condensate in asthmatic children during acute exacerbation and after treatment Respiration 2012 84 291 298 10.1159/000341969 23018317

[B20] Corradi M Zinelli C Caffarelli C Exhaled breath biomarkers in asthmatic children Inflamm Allergy Drug Targets 2007 6 150 159 10.2174/187152807781696437 17897051

[B21] Zinelli C Caffarelli C Strid J Jaffe A Atherton DJ Measurement of nitric oxide and 8-isoprostane in exhaled breath of children with atopic eczema Clin Exp Dermatol 2009 34 607 612 10.1111/j.1365-2230.2008.03142.x 19508477

[B22] Bousquet J Neukirch F Bousquet PJ Gehano P Klossek JM Le Gal M Allaf B Severity and impairment of allergic rhinitis in patients consulting in primary care J Allergy Clin Immunol 2006 117 158 162 10.1016/j.jaci.2005.09.047 16387600

[B23] Miraglia Del Giudice M Marseglia A Leonardi S La Rosa M Salpietro C Brunese FP Arrigo T Perrone L Allergic rhinitis and quality of life in children Int J Immunopathol Pharmacol 2011 24 Suppl 4 25 28 22032783 10.1177/03946320110240s406

[B24] Tosca MA Riccio AM Marseglia GL Caligo G Pallestrini E Ameli F Mira E Castelnuovo P Pagella F Ricci A Ciprandi G Canonica GW Nasal endoscopy in asthmatic children: assessment of rhinosinusitis and adenoiditis incidence, correlations with cytology and microbiology Clin Exp Allergy 2001 31 609 615 10.1046/j.1365-2222.2001.01057.x 11359430

[B25] Compalati E Ridolo E Passalacqua G Braido F Villa E Canonica GW The link between allergic riniti and asthma: the united airways disease Expert Rev Clin Immunol 2010 6 413 423 10.1586/eci.10.15 20441427

[B26] Ciprandi G Cirillo I Klersy C Marseglia GL Vizzaccaro A Pallestrini E Tosca M Role of FEF25–75 as an early marker of bronchial impairment in patients with seasonal allergic rhinitis Am J Rhinol 2006 20 641 647 10.2500/ajr.2006.20.2914 17181110

[B27] Ciprandi G Tosca MA Castellazzi AM Cairello F Salpietro C Arrigo T Miraglia Del Giudice M FEF(25–75) might be a predictive factor for bronchial inflammation and bronchial hyperreactivity in adolescents with allergic rhinitis Int J Immunopathol Pharmacol 2011 24 Suppl 4 17 20 22032781 10.1177/03946320110240S404

[B28] Ciprandi G Cuppari C Salpietro AM Tosca AM Rigoli L Grasso L La Rosa M Marseglia GL Del Giudice MM Salpietro C Serum IL-23 Strongly and Inversely Correlates with FEV(1) in Asthmatic Children Int Arch Allergy Immunol 2012 159 183 186 22678234 10.1159/000336418

[B29] Ciprandi G Capasso M Tosca M Salpietro C Salpietro A Marseglia G La Rosa M A forced expiratory flow at 25-75% value <65% of predicted should be considered abnormal: a real-world, cross-sectional study Allergy Asthma Proc 2012 33 e5 e8 10.2500/aap.2012.33.3524 22370528

[B30] Simons FER Allergic rhinobronchitis: the asthma–allergic rhinitis link J Allergy Clin Immunol 1994 104 534 540 10482824 10.1016/s0091-6749(99)70320-9

[B31] Ciprandi G Cirillo I The lower airway pathology of rhinitis J Allergy Clin Immunol 2007 119 1557 1558 10.1016/j.jaci.2007.01.048 17368528

[B32] Valero A Pereira C Loureiro C Martinez-Cocera C Murio C Rico C Palomino R Davila I Interrelationship between skin sensitization, rhinitis, and asthma in patients with allergic rhinitis: a study of Spain and Portugal J Investig Allergol Clin Immunol 2009 19 167 172 19610258

[B33] Staikunienè J Vaitkus S Marija L Ryskiene S Association of chronic rhinosinusitis with nasal polyps and asthma: clinical and radiological features, allergy and inflammation markers Medicina (Kaunas) 2008 44 257 265 18469501

[B34] Jani AL Hamilos DL Current thinking on the relationship between rhinosinusitis and asthma J Asthma 2005 42 1 7 10.1081/JAS-200044744 15801321

[B35] Peroni DG Piacentini GL Ceravolo R Boner AL Difficult asthma: possible association with rhinosinusitis Pediatr Allergy Immunol 2007 18 S25 S27 10.1111/j.1399-3038.2007.00628.x 17767603

[B36] Bresciani M Paradis L Des Roches A Vernhet H Vachier I Godard P Bousquet J Chanez P Rhinosinusitis in severe asthma J Allergy Clin Immunol 2001 107 73 80 10.1067/mai.2001.111593 11149994

[B37] Meltzer E Szwarcberg J Pill MW Allergic rhinitis, asthma and rhinosinusitis: diseases of the integrated airway J Manag Care Pharm 2004 10 310 317 15298529 10.18553/jmcp.2004.10.4.310 PMC10438157

[B38] Steinke JW The relationship between rhinosinusitis and asthma sinusitis Curr Allergy Asthma Rep 2006 6 495 501 10.1007/s11882-006-0027-2 17026875

[B39] Ponte EV Franco R Nascimento HF Souza-Machado A Cunha S Barreto ML Naspitz C Cruz AA Lack of control of severe asthma is associated with co-existence of moderate-to-severe rhinitis Allergy 2008 63 564 569 10.1111/j.1398-9995.2007.01624.x 18394130

[B40] Crystal-Peters J Neslusan C Crown WH Torres A Treating allergic rhinitis in patients with comorbid asthma: the risk of asthma-related hospitalizations and emergency department visits J Allergy Clin Immunol 2002 109 57 62 10.1067/mai.2002.120554 11799366

[B41] Nathan RA Yancey SW Waitkus-Edwards K Prillaman BA Stauffer JL Philpot E Dorinsky PM Nelson HS Fluticasone propionate nasal spray is superior to montelukast for allergic rhinitis while neither affects overall asthma control Chest 2005 128 1910 1920 10.1378/chest.128.4.1910 16236835

[B42] Marseglia GL Caimmi D Pagella F Matti E Labó E Licari A Salpietro A Pelizzo G Castellazzi AM Adenoids during childhood: the facts Int J Immunopathol Pharmacol 2011 24 Suppl 4 1 5 22032778 10.1177/03946320110240S401

[B43] La Rosa M Lionetti E Leonardi S Salpietro A Bianchi L Salpietro C Miraglia Del Giudice M Ciprandi G Marseglia GL Specific immunotherapy in children: the evidence Int J Immunopathol Pharmacol 2011 24 Suppl 4 69 78 22032790 10.1177/03946320110240S413

[B44] Caffarelli C Cardinale F Povesi C Chinellato I Sterpeto Loffredo MB Optimal duration of SLIT Int J Immunopathol Pharmacol 2009 22 17 22 19944005

[B45] Cardinale F Chinellato I Caimmi S Peroni DG Franceschini F Miraglia Del Giudice M Bernardini R Perioperative period: immunological modifications Int J Immunopathol Pharmacol 2011 24 Suppl 3 3 12 10.1177/03946320110240s302 22014920

[B46] Caimmi S Caimmi D Bernardini R Caffarelli C Crisafulli G Pingitore G Marseglia GL Perioperative anaphylaxis: epidemiology Int J Immunopathol Pharmacol 2011 24 Suppl 3 21 26 10.1177/03946320110240s304 22014922

[B47] Caffarelli C Stringari G Pajno GB Peroni DG Franceschini F Dello Iacono I Bernardini R Perioperative allergy: risk factors Int J Immunopathol Pharmacol 2011 24 Suppl 3 27 34 10.1177/03946320110240s305 22014923

[B48] Leonardi S Miraglia Del Giudice M La Rosa M Bellanti JA Atopic disease, immune system, and the environment Allergy Asthma Proc 2007 28 410 417 10.2500/aap.2007.28.2954 17883908

